# Routine mapping of Fusarium wilt resistance in BC_1_ populations of *Arabidopsis thaliana*

**DOI:** 10.1186/1471-2229-13-171

**Published:** 2013-10-30

**Authors:** Andrew C Diener

**Affiliations:** 1 Department of Molecular, Cell and Developmental Biology, University of California, Terasaki Life Sciences Building, 610 Charles E. Young Drive East, Los Angeles, CA 90095, USA

**Keywords:** *Arabidopsis thaliana*, *Fusarium oxysporum*, Quantitative disease resistance, QTL mapping, Crossover interference

## Abstract

**Background:**

Susceptibility to Fusarium wilt disease varies among wild accessions of *Arabidopsis thaliana*. Six *RESISTANCE TO FUSARIUM OXYSPORUM* (*RFO*) quantitative trait loci (QTLs) controlling the resistance of accession Columbia-0 (Col-0) and susceptibility of Taynuilt-0 to *Fusarium oxysporum* forma specialis *matthioli* (FOM) are detected in a recombinant population derived from a single backcross of the F_1_ hybrid (BC_1_). In particular, the *RFO1* QTL appears to interact with three other loci, *RFO2*, *RFO4* and *RFO6*, and is attributed to the gene At1g79670.

**Results:**

When resistance to FOM was mapped in a new BC_1_ population, in which the loss-of-function mutant of At1g79670 replaced wild type as the Col-0 parent, *RFO1*’s major effect and *RFO1*’s interaction with *RFO2*, *RFO4* and *RFO6* were absent, showing that At1g79670 alone accounts for the *RFO1* QTL. Resistance of two QTLs, *RFO3* and *RFO5*, was independent of *RFO1* and was reproduced in the new BC_1_ population. In analysis of a third BC_1_ population, resistance to a second pathogen, *F. oxysporum* forma specialis *conglutinans* race 1 (FOC1), was mapped and the major effect locus *RFO7* was identified.

**Conclusions:**

Natural quantitative resistance to *F. oxysporum* is largely specific to the infecting forma specialis because different *RFO* loci were responsible for resistance to FOM and FOC1. The mapping of quantitative disease resistance traits in BC_1_ populations, generated from crosses between sequenced *Arabidopsis* accessions, can be a routine procedure when genome-wide genotyping is efficient, economical and accessible.

## Background

Fusarium wilt of *Arabidopsis thaliana* is an ideal pathosystem for mapping, identifying and characterizing genes responsible for host resistance to vascular wilt fungi. *A. thaliana*, which is the preeminent subject of plant molecular genetic and genomic studies, is susceptible to infection by three phylogenetically-distinct pathogenic forms, or formae speciales, of the soil-borne fungus *Fusarium oxysporum*[1,2]. In the field, *F. oxysporum* forma specialis *conglutinans* (FOC), *F. oxysporum* forma specialis *raphani* and *F. oxysporum* forma specialis *matthioli* (FOM) are isolated from diseased *Brassica* species, radish (*Raphanus sativus*) and garden stock (*Matthioli incana*), respectively [3]. Fusarium wilt of *A. thaliana* recapitulates the development of disease symptoms in field hosts [1].

The response of different accessions of *A. thaliana* to different formae speciales varies from complete resistance to ready susceptibility [1]. For example, the standard laboratory accession Columbia-0 (Col-0) is completely resistant to FOM but expresses only partial resistance to FOC1. Taynuilt-0 (Ty-0), on the other hand, is susceptible to FOM but also expresses partial resistance to FOC1.

Two strategies are used to map genes responsible for phenotypic variation in populations [4-6]. When the population of interest is wild and results from an indeterminate number of undefined crosses, a genome-wide association (GWA) study uses evidence of linkage disequilibrium to associate sequence polymorphisms within or near the genes responsible for the trait. Enabling GWA studies in the plant *A. thaliana* is the primary motivation for the 1001 Genomes Project, which has generated whole genome sequence for hundreds of wild accessions of *A. thaliana*[7,8]. Indeed, the detection of functional sequence diversity in *A. thaliana* using GWA is reported [9,10]. However, GWA studies rarely detect more than a modest fraction of the sequence diversity responsible for variation in existing populations of plant and animal species [5,9,11].

Genetic linkage may be used to map the genes associated with a trait to chromosomal intervals. However, this approach requires that the studied population is derived from controlled crosses between defined parents; and, only the genetic diversity distinguishing the parents of crosses is detected. Nevertheless, linkage analysis has been a powerful and successful approach for detecting and defining the genes responsible for complex traits in *A. thaliana*[12].

With plant species that readily inbreed, such as *A. thaliana*, recombinant inbred (RI) populations are almost exclusively used to map and define genetic loci underlying natural traits [12,13]. RI populations in their simplest form originate from an outcross between parents with dissimilar genotypes. Unique recombinant genotypes of the parents are captured in dozens to hundreds of RI lines that result from propagating individual F_2_ offspring by self-fertilization and single-seed descent. After several filial generations of inbreeding, RI progeny become largely homozygous and thus true-breeding RI lines. However, the effort to propagate and curate an RI population without introducing selection represents a substantial investment in time and effort before QTL analysis begins. The effort to generate an RI population is offset by the fact that RI lines are immortal and can be retested innumerable times and reused in separate studies but need to be genotyped just once. There are now dozens of published RI populations from crosses between wild accessions of *A. thaliana*[12,14-16]. Recently, a technique for generating haploid *A. thaliana* has made the generation of doubled haploid lines possible [17]. Like RI lines, doubled haploids are homozygous and thus immortal but require fewer generations to create.

Other mating strategies generate recombinant mapping populations in less time and with less effort than it takes to generate RI lines. In particular, BC_1_ populations are generated from crosses in two successive generations. An initial outcross between parental genotypes produces the F_1_ hybrid, which is then backcrossed to its recurrent parent. Each resulting BC_1_ hybrid inherits a set of non-recombinant chromosomes from the recurrent parent and a set of recombinant chromosomes from the F_1_ hybrid. Because crossovers resulting from single meioses can be unambiguously assigned to recombinant chromosomes, the BC_1_ mating scheme is often used to generate a model population for the evaluation of novel approaches to QTL analysis [18-20]. In addition, backcrossing is a common feature in traditional breeding schemes that seek to introgress new traits into elite crop varieties [21].

The appeal of BC_1_ populations is undermined by the need for extensive genotyping, and very few studies of natural traits in *A. thaliana* have used BC_1_ populations for genome-wide mapping [1,12,22]. Because each BC_1_ hybrid possesses a unique recombinant genotype, it is necessary to genotype each tested BC_1_ hybrid genome-wide. Without whole genome sequence information for the parents of a BC_1_ population, the discovery of sequence polymorphism and their development into an appropriate set of DNA markers for genome-wide mapping is a time-consuming and laborious process.

Nevertheless, prior genetic analysis of a BC_1_ population shows that the qualitative resistance of Col-0 to FOM is a polygenic trait [1]. Six *RFO* QTLs, accounting for the resistance of Col-0 and susceptibility of Ty-0, segregate in a population generated by crossing Col-0 and Ty-0 and then backcrossing the resistant F_1_ hybrid to its susceptible parent Ty-0. Among *RFO* loci, *RFO1* has the strongest association with resistance to FOM. *RFO1* also appears to interact with three other *RFO* loci, namely *RFO2*, *RFO4* and *RFO6*, because the three interacting loci have significant association only when recombinant BC_1_ hybrids also inherit the Col-0 allele of *RFO1* (*RFO1-C*). *RFO2* is a receptor-like protein (RLP) gene that is homologous to the PSY1 peptide receptor gene, *PSY1R*[23]. The *RFO1*-linked gene At1g79670 is named *RFO1* because the Col-0 sequence of At1g79670, as a transgene, enhances the resistance of Ty-0, and the loss-of-function allele of At1g79670 (*rfo1*) compromises the resistance of Col-0 [1]. At1g79670 is a member of the wall-associated kinase-like kinase subfamily of receptor-like kinase (RLK) genes.

Here, I map Fusarium wilt resistance in two new BC_1_ populations (i) to address whether At1g79670 alone is responsible for resistance attributed to the *RFO1* QTL, including interactions with *RFO2*, *RFO4* and *RFO6*, and (ii) to examine whether the same or different *RFO* QTLs mediate resistance to different formae speciales of *F. oxysporum*. In doing so, I present a methodology for genome-wide genotyping that makes the mapping of complex quantitative traits a routine procedure. Importantly, because whole genome sequence is now available for most studied accessions, the same approach could be applied to crosses between any pair of *Arabidopsis* accessions.

## Results

### Resistance to FOM in *rfo1*

In prior mapping of resistance to FOM, *RFO1* was the most significant of six *RFO* loci in *A. thaliana*, and *RFO1* was epistatic to, or enhanced the resistance of, three other *RFO* loci [1]. In theory, the *RFO1* QTL could represent one gene or multiple genes. To appreciate whether At1g79670 is responsible for all or part of the resistance attributed to the *RFO1* QTL, resistance to FOM was mapped in a new BC_1_ population that included *rfo1*, which is a loss-of-function allele of At1g79670 resulting from a T-DNA insertion in and deletion of coding sequence in the Col-0 genetic background [1,24]. The same crossing scheme that generated the original Col-0 and Ty-0 (C-T) BC_1_ population, was used to generate the new *rfo1* and Ty-0 (*r*-T) BC_1_ population with the exception that *rfo1* replaced wild type as the Col-0 parent: Crossing *rfo1* and Ty-0 produced the F_1_ hybrid that was then backcrossed to Ty-0. Differences in quantitative resistance in the new *r*-T and original C-T populations would include the contribution of At1g79670.

**Figure 1:**
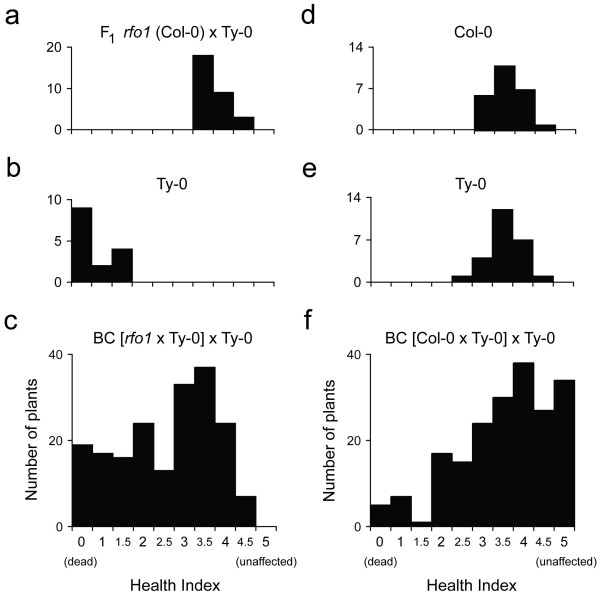
**Health of *F. oxysporum*-infected plants.** Health index (HI) scores of FOM-infected plants at 18 dpi: Col-0/Ty-0 F_1_ hybrids **(a)**, Ty-0 **(b)** and BC_1_ hybrids in *r*-T population **(c)**; and, HI scores of FOC1-infected plants at 16 dpi: Col-0 **(d)**, Ty-0 **(e)**, and BC_1_ hybrids in C-T population **(f)**. At the extremes, plants were dead (HI = 0) or unaffected (HI = 5.0).

As in the C-T population, resistance to FOM segregated in the *r*-T population as a polygenic trait, and most BC_1_ hybrids exhibited resistance that was intermediate to that of either parent [1]. Wilt disease in the F_1_ hybrid, Ty-0 parent and 190 BC_1_ hybrids was evaluated using a health index (HI), an ordinal scale from 0 (dead) to 5 (unaffected), described in Methods. At 18 days post infection (dpi), a broad distribution of HI scores registered the breadth of disease resistance among BC_1_ hybrids and presumably the diversity of resistance genotypes (Figure 1c). In contrast, the parents were consistently either resistant or susceptible. Most F_1_ hybrids (Figure 1a) as well as a minority of BC_1_ hybrids (Figure 1c) exhibited only mild symptoms (with a HI score > 3); and, at the opposite extreme, most of the Ty-0 parents (60 percent, Figure 1b) as well as 10 percent of BC_1_ hybrids were dead (Figure 1c). Thus, segregation of resistance among BC_1_ hybrids was inconsistent with monogenic inheritance as a single locus would have given a 1:1 segregation ratio in the backcross, *i.e.* one plant as resistant as the F_1_ hybrid to one plant as susceptible as the Ty-0 parent.

### Genome-wide linkage of 40 CHR markers

To expedite the mapping of resistance, methodology to genotype BC_1_ hybrids was developed with efficiency and economy in mind. Previously, *RFO* QTLs were mapped in the C-T population using the genome-wide genotype of 24 SSLP markers distributed over the five chromosomes of *A. thaliana*[1]. However, genotyping one SSLP in one BC_1_ hybrid from one PCR sample is a prohibitive bottleneck in analysis. For instance, if the same 24 SSLPs were used to genotype the 190 FOM-infected BC_1_ hybrids in the *r*-T population, the effort would entail processing no fewer than 4,560 PCR samples. Instead, as described in Methods, the genome-wide genotype of 40 marker loci in each BC_1_ hybrid was obtained from just three multiplex PCR samples. In comparison to genotyping with SSLPs, the new approach gave genome-wide genotypes of BC_1_ hybrids that were comprised of two-thirds more markers and obtained in one-eighth as many PCR samples.

**Figure 2:**
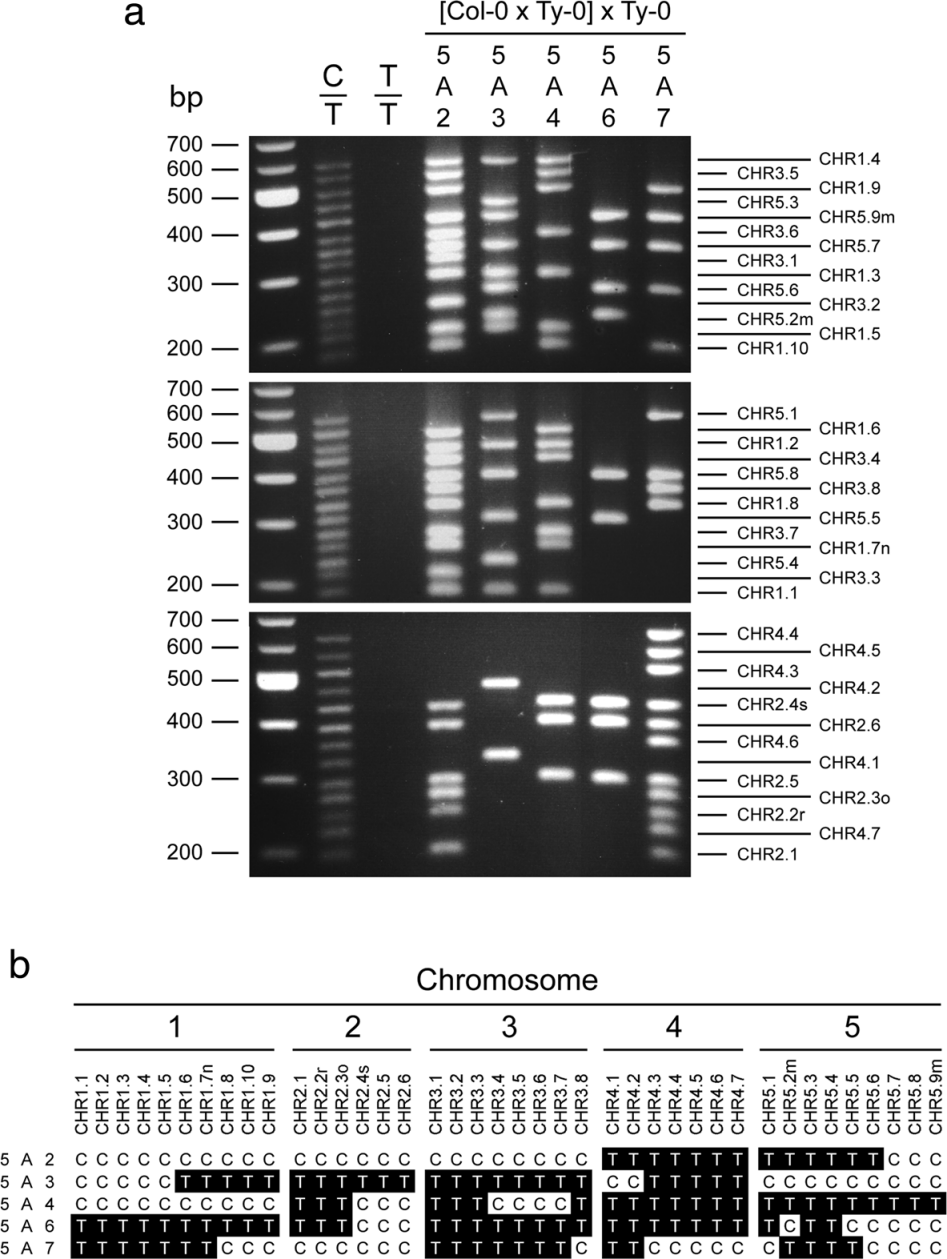
**Genome-wide genotyping with CHR markers. (a)** Multiplex PCR products for 40 Col-0-specific dominant markers were size-separated by agarose-gel electrophoresis and stained with ethidium bromide. Sizes in basepairs (bp) for the DNA ladder (leftmost lane) are at left. Marker DNA was PCR-amplified from the Col-0 and Ty-0 F_1_ hybrid (C/T), accession Ty-0 (T/T) and C-T BC_1_ hybrids, 5A2, 5A3, 5A4, 5A6 and 5A7. Lines to the right indicate the expected positions of marker bands. Markers are named CHR*x*.*n*, where *x* is the chromosome and *n* is the relative position on the chromosome. **(b)** Genotypes of markers in five BC_1_ hybrids are from banding phenotypes in **(a)**. Markers are ordered with respect to their position on chromosomes. Genotype C/T (C) is shown with black on white type, and genotype T/T (T) is white on black type.

The phenotype of the 40 CHR markers was dominant, and primer pairs for CHR markers directed PCR amplification of marker sequence from Col-0 DNA and not from Ty-0 DNA (Figure 2a). DNA products corresponding to as many as 14 markers were amplified in a single multiplex PCR sample and then separated by size using standard agarose gel electrophoresis, as shown for the three multiplex PCR samples of five representative BC_1_ hybrids in Figure 2a. Because BC_1_ hybrids were either Col-0/Ty-0 (C/T) or Ty-0/Ty-0 (T/T) at any locus, genotypes were scored according to whether PCR-amplified marker DNA was present (C) or absent (T), respectively (Figure 2b).

Genetic linkage between CHR markers in both *r*-T and C-T populations was consistent with the proximity and order of marker sequences in the *Arabidopsis* reference genome (version TAIR10, http://www.arabidopsis.org). Genome-wide genetic maps corresponding to recombination frequencies in *r*-T and C-T populations are shown in Additional file 1: Figure S1 and Additional file 2: Figure S2, respectively. In the *r*-T population, marker intervals had mean, median and total genome distances of 15.8 centiMorgan (cM), 12.9 cM and 551 cM, respectively, while individual marker intervals ranged from 4.8 cM to 27.1 cM. (See Additional file 3: Table S1 for recombination frequencies and genetic distances of all intervals). In the original C-T population, 39 dominant markers and one SSLP marker (in place of the linked CHR2.4 marker) had mean, median and total genome distances of 14.1 cM, 14.5 cM, and 516 cM, respectively (See Additional file 4: Table S2 for recombination frequencies and genetic distances of all intervals).

### Reliability of CHR markers

There was concern that dominant CHR markers would not be as reliable as codominant SSLP markers. The absence of marker DNA, which is the phenotype of genotype T/T, could be the false negative result of insufficient PCR amplification of Col-0 DNA from genotype C/T in which case a genotype of C/T would be miscalled as T/T. The codominant SSLPs, on the other hand, were safeguarded from false negative miscalls because marker primers direct the amplification of Ty-0 DNA in all samples, confirming that PCR was productive.

Results with SSLPs and CHR markers were compared in the C-T population. As expected, half of genotypes at SSLP markers (50.4 percent with a standard deviation of 3.4 percent) and half of genotypes at CHR markers (50.9 percent with a standard deviation of 3.0 percent) were T/T, so neither codominant SSLPs nor the Col-0-specific CHR markers were prone to give an excess of T/T.

**Figure 3:**
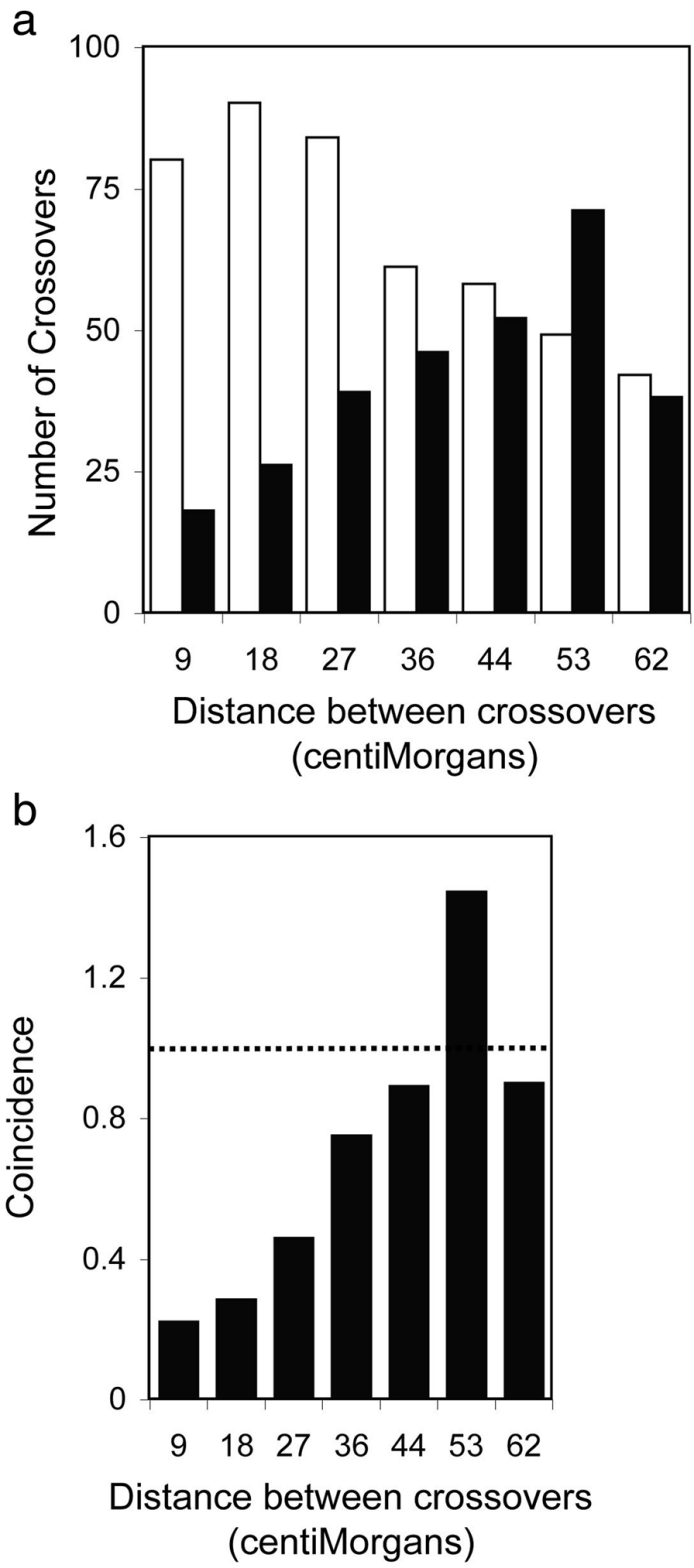
**Crossover interference. (a)** Among 63 markers in 234 FOM-infected C-T BC_1_ hybrids, the number of expected (open bar) or observed (filled bar) crossovers is shown at the indicated distance between crossovers. **(b)** Coincidence of crossovers is the observed crossover frequency in two marker intervals divided by expected crossover frequency. Observed and expected frequencies are equivalent at 1 (dashed line). Crossovers are separated by number of markers (estimated distance in cM): 1 (9), 2 (18), 3 (26), 4 (35), 5 (44), 6 (53) and 7 (62). The estimated distance between crossovers is the mean distance between adjacent markers (8.7 cM) times the number of markers.

The reliability of dominant markers was further scrutinized by examining recombination in a dataset that combined the genotypes of 39 dominant markers and 24 SSLP markers in the C-T population. Miscalled marker genotypes would exaggerate the number of instances of crossovers in adjacent marker intervals because tightly linked markers usually share the same genotype. The mean recombination frequency in intervals separating the 63 markers was 8.7 percent, so pairs of adjacent intervals were expected to have crossover events only once or twice among 234 BC_1_ hybrids. A miscalled genotype would appear to be flanked, in most cases, by markers with opposite genotype and thus by intervals with spurious crossovers. However, instead of having an excess of adjacent double crossovers, the combined marker dataset had a clear deficit of linked double crossovers (Figure 3a). A total of 80 double crossovers were predicted from the sum of the products of recombination frequencies in adjacent intervals, whereas crossovers in adjacent intervals were observed in just 18 instances. Importantly, double crossovers flanked a similar proportion of dominant markers (10) and SSLPs (8). Thus, dominant markers were no more likely than SSLPs to have genotypes that were different from the genotypes of both flanking markers. In addition, the number of crossovers in two intervals was expected to decline as the number of marker intervals separating crossovers increased, whereas the observed number of double crossovers increased with separation of crossover events (Figure 3a).

### Crossover interference

The deficiency of linked crossovers was explained by crossover interference, which is observed in *A. thaliana*[25]. Coincidence, a measure of crossover interference, is defined as the observed frequency of crossovers in two marker intervals divided by the product of recombination frequencies in the same intervals [26]. Positive interference has a coincidence value of less than one and indicates that a crossover in one interval inhibits crossover in the other interval. In Figure 3b, positive interference was observed when crossovers were separated by less than 36 cM. Coincidence values near one or greater than one indicate no interference or negative interference, respectively. A transitional negative interference, which is a common observation when positive interference is present, was apparent when crossovers were separated by roughly 53 cM in Figure 3b [26]. Moreover, clear deficiencies of linked double crossovers were observed in all BC_1_ populations examined here. Only 19 to 33 percent of expected double crossovers in adjacent marker intervals were in fact observed in the five *Arabidopsis* chromosomes. (For chromosomal distribution of expected and observed double crossovers, see Additional file 5: Table S3.)

### No *RFO1* QTL without At1g79670

In the *r*-T population, association of resistance at CHR markers was evaluated using the Mann-Whitney rank sum test as previously described in [1]. Briefly, BC_1_ hybrids in the *r*-T population were ranked, from most susceptible to most resistant, according to HI scores. At each marker, a standardized statistic *Z* enumerated the separation of ranks of BC_1_ hybrids that were C/T and T/T, and the sign and magnitude of *Z* indicated the direction and strength of genetic association. Specifically, resistance was found to have significant correlation with genotype C/T when *Z* was greater than 3.3 and with T/T when *Z* was less than -3.3 (when *p* < 0.05, according to permutation tests).

**Figure 4:**
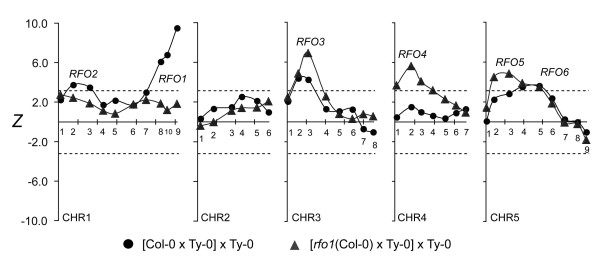
**Association of resistance to FOM.** In FOM-infected C-T (circle) and *r*-T (diamond) BC_1_ populations, test statistic *Z* correlates resistance and marker genotype. Lines connect values of linked markers. Marker placement on *x*-axis corresponds to nucleotide position in TAIR reference sequence of five Arabidopsis chromosomes (CHR1 through CHR5): Ticks are spaced by 20 Megabps. Markers are named CHR*x*.*n*, where *x* and *n* indicate chromosome and relative marker position, and are labeled above or below the x-axis. Dashed lines indicate the threshold values of *Z* below which negative values, or above which positive values, are attained with *p* < 0.05.

**Figure 5:**
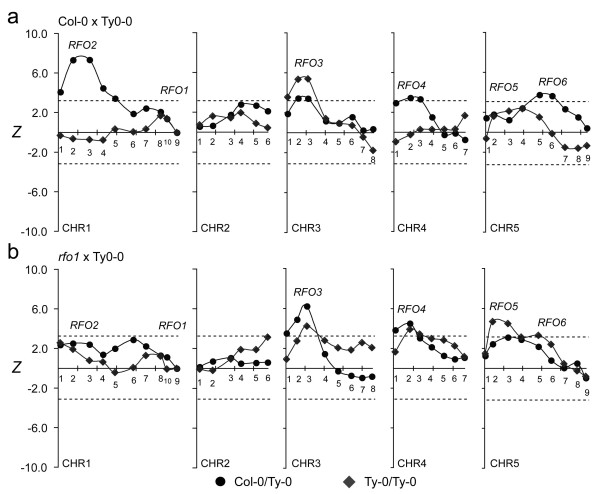
***RFO1-*conditioned resistance to FOM.** Subpopulations of FOM-infected **(a)** C-T and **(b)***r*-T BC_1_ populations are conditioned by whether BC_1_ hybrids inherited *RFO1-C* (C/T, circle) or not (T/T, diamond). See Figure 4 for description of plot details.

In the *r*-T population, no QTL with major effect was detected on chromosome 1, though both *RFO1* and *RFO2* are located on chromosome 1 and make substantial contribution to resistance in the C-T population (Figure 4) [1]. In the *r*-T population, the correlation of resistance with the Col-0 alleles of *RFO1*- and *RFO2*-linked markers, respectively CHR1.9 and CHR1.3, lacked statistical significance (Figure 4). Thus, *rfo1* abolished the major contributions of *RFO1* and *RFO2*. In the C-T population, *RFO2*’s strong association with resistance among plants that are C/T at *RFO1* is absent among plants that were T/T (Figure 5a) [1]. In the *r*-T population, *RFO2*-linked markers had insignificant association with resistance whether BC_1_ hybrids were C/T or T/T at *RFO1* (Figure 5b).

*rfo1* also suppressed the apparent interactions between *RFO1* and either the (CHR5.6-linked) *RFO6* or (CHR4.2-linked) *RFO4*. From prior work, resistance is associated with two loci on chromosome 5: *RFO5* gives resistance that is independent of *RFO1* while *RFO6* is only evident among BC_1_ hybrids that are also C/T at *RFO1* (Figure 5a) [1]. In the *r*-T population, CHR5.6 lacked significant association with resistance among BC_1_ hybrids with or without *RFO1-C* (Figure 5b). Similarly, an apparent interaction between *RFO1* and (CHR4.2-linked) *RFO4* was not evident in the *r*-T population, whereas significant association of resistance at *RFO4* in the C-T population is evident only among plants that also have *RFO1-C* (Figure 5a) [1]. In the *r*-T population, marker CHR4.2 was associated with a major QTL without regard to the genotype of *RFO1* (Figure 5b).

As previously observed, *RFO3* and *RFO5* expressed resistance that was independent of *RFO1*[1]. In fact, *RFO3* and *RFO5* had stronger correlation with resistance in the *r*-T population than in the C-T population –compare peak *Z* values at *RFO3-*linked (CHR3.3) and *RFO5*-linked (CHR5.3) in Figure 4. Excluding *RFO3*, *RFO4* and *RFO5*, there was no other significant association with resistance to FOM.

### *RFO* QTLs are pathogen-specific

To examine whether the same or different *RFO* loci provided resistance to different *F. oxysporum* pathogens, resistance was investigated in a third BC_1_ population that was instead infected with FOC1. The HI scores of 200 FOC1-infected BC_1_ hybrids and the two parental accessions, Col-0 and Ty-0, at 16 dpi are shown in Figure 1d, e and f, respectively. Both parental accessions exhibited partial resistance to FOC1 and had median HI scores of 3.5 (Figure 1d, e). BC_1_ hybrids exhibited a broader range of symptom severity than their parents (Figure 1f): 17 percent of BC_1_ hybrids were unaffected (HI = 5.0) while all parents exhibited at least mild symptoms; and, 15 percent of BC_1_ hybrids exhibit more severe symptoms that either parent (HI < 2.0). Thus, a third of FOC1-infected BC_1_ hybrids expressed an extreme phenotype that was not seen in either parent.

### *RFO7* confers resistance to FOC1

A genome-wide genetic map derived from the recombination frequencies between CHR markers in the FOC1-infected C-T population was consistent with the order of marker sequences in the TAIR10 reference genome (See Additional file 6: Figure S3 for the genome-wide genetic map). Intervals between markers ranged from 4.0 to 24.9 cM, and mean, median and total genome distances were 13.5, 12.8 and 472 cM, respectively (See Additional file 7: Table S4 for recombination frequencies and genetic distances of all marker intervals).

**Figure 6:**
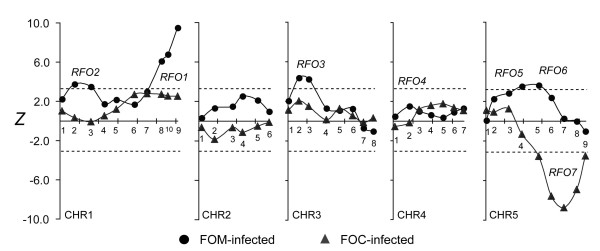
**Association of resistance to FOC1.** In FOM-infected (circles) and FOC1-infected (triangles) C-T populations, test statistic *Z* enumerates the correlation of resistance and marker genotype, and lines connect linked values. See Figure 4 for description of plot details.

**Figure 7:**
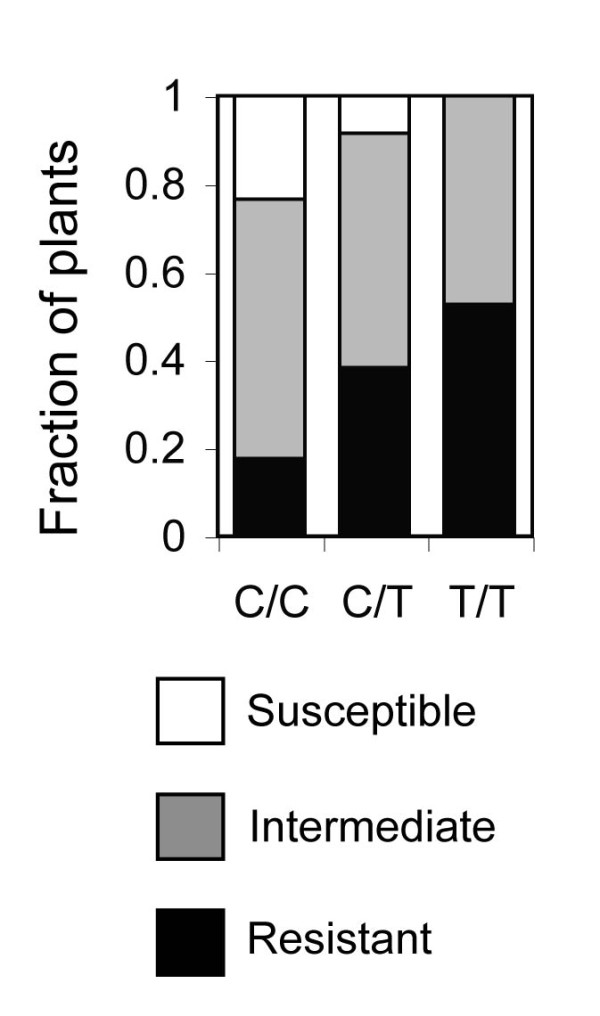
**Resistance to FOC1 at *RFO7.*** In F_2_ progeny of Col-0 and Ty-0, wilt resistance cosegregates with *RFO7*-linked SSLP CIW9. F_2_ heterozygotes (C/T, *n* = 37) and homozygotes (C/C, *n* = 17; or, T/T, *n* = 19) were resistant (HI scores of 4 or 5, open bar) or susceptible (HI score of 0 or 1, black-filled bar) or had intermediate resistance (HI scores of 2 or 3, gray-filled bar). M-W test indicates that symptom severity in C/C and C/T (*p* = 0.005) or in C/C and T/T (*p* = 0.0006) was dissimilar.

Association with resistance to FOC1 was evaluated at 40 CHR markers. For the sake of comparison, *Z* statistics at markers in FOC1-infected and FOM-infected C-T populations are juxtaposed in Figure 6. A single major effect QTL at marker CHR5.7 (*Z* = -8.77) associated genotype T/T with strong resistance to FOC1. Because all previous *RFO* QTLs correlated resistance with genotype C/T and CHR5.7 was not previously associated with resistance, this QTL was new and was named *RFO7*. Among F_2_ offspring of Col-0 and Ty-0, genotype C/C at the *RFO7*-linked SSLP CIW9 was more susceptible to FOC1 than genotype C/T, indicating that Col-0 and Ty-0 alleles of *RFO7* express incomplete dominance (Figure 7).

**Figure 8:**
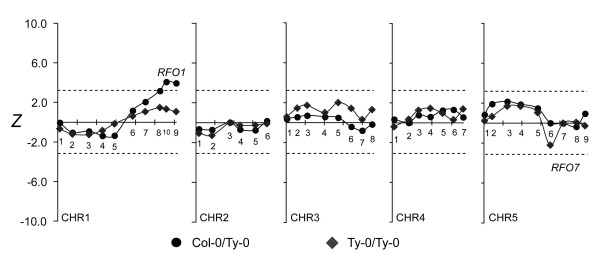
***RFO7*-conditioned resistance to FOC1.** Subpopulations of FOC1-infected C-T BC_1_ population were conditioned by whether BC_1_ hybrids inherited *RFO7-C* (C/T, circle) or not (T/T, diamond). See Figure 4 for description of plot details.

Previously, *RFO1* was shown to confer resistance to FOC1 as well as FOM [1]. In the FOC1-infected C-T population, resistance associated with *RFO1-*linked CHR1.9 had questionable significance (*Z* = 2.54, *p* = 0.28). However, among BC_1_ hybrids that were heterozygotes (C/T) at CHR5.7, which minimized the contribution of *RFO7*, the association of resistance with *RFO1* was significant (Figure 8).

## Discussion

It was conceivable that more than one gene might be responsible for different aspects of the *RFO1* QTL. However, QTL analysis that included *rfo1* was consistent with the simplest explanation: A single gene was responsible for the major effect of *RFO1* and also for apparent interactions with *RFO2*, *RFO4* and *RFO6*. *RFO2* and *RFO6* were undetected in the *r*-T population while the resistance of *RFO4*, which was dependent on *RFO1-C* in the C-T population, was independent of *RFO1* in the *r*-T population.

Why *RFO4*, which only attained significance with *RFO1-C* in the C-T population, was a major QTL in the *r*-T population lacking *RFO1-C* is difficult to explain. Possibly, the expression of *RFO4* was influenced by subtle differences in the progression of wilt disease or environmental factors as the C-T and *r*-T populations were similarly infected on separate occasions. Also, the parents, which were nominally from the same Col-0 and Ty-0 accessions, might have been genetically (or epigenetically) dissimilar as separate crosses generated the two populations.

Overall, results obtained from independent FOM-infected populations were consistent, and QTLs in the new *r*-T population were coincident with the previously detected *RFO3*, *RFO4* and *RFO5* in the C-T population. In fact, the association of resistance at the three QTLs appeared stronger in the *r*-T population. Col-0, which was the source of resistance, rarely exhibits wilt symptoms when infected with FOM and presumably expresses more than sufficient resistance. As symptom severity is difficult to discriminate among the more resistant plants, loss of *RFO1-C* in the *r*-T population undoubtedly improved the evaluation of disease in BC_1_ hybrids and thus the detection of *RFO* QTLs.

The map position and source of QTLs detected in FOC1- and FOM-infected populations suggest that quantitative resistance to *F. oxysporum* is predominantly specific to the infecting forma specialis. Remarkably, resistance to FOC1 was strongly associated with a single new QTL, *RFO7*, though Col-0 and Ty-0 expressed similar partial resistance to FOC1. In addition to *RFO7*, a region on chromosome 1 had a marginal association with resistance, and *RFO1*-linked markers did attain significant association when BC_1_ hybrids were heterozygous for *RFO7*. However, previous work clearly shows that *rfo1* and transgenic *RFO1* affect resistance to both FOC1 and FOM. Thus, while *RFO1* may have a non-specific role in resistance to the three crucifer-infecting *formae speciales*, it appears that *RFO1* also has a much stronger, specific effect on resistance to FOM.

Only dominant traits from the donor parent are expressed in a BC_1_ population, so *RFO* alleles of Col-0 that were recessive to alleles of Ty-0 would not be detected. Nevertheless, the strong resistance of F_1_ hybrids, Col-0/Ty-0 and *rfo1*/Ty-0, suggests that resistance to *F. oxysporum* is in large part a dominant trait. Resistance associated with *RFO7* was confirmed in F_2_ progeny, and positional cloning has identified single genes that are responsible for three *RFO* QTLs, *RFO1*, *RFO2* and *RFO3*[1,23,27]. Of the four confirmed *RFO* loci, three QTLs, *RFO1*, *RFO3* and *RFO7*, express incomplete dominance.

Nucleotide sequences of both resistant and susceptible alleles of *RFO1*, *RFO2* and *RFO3* encode apparently functional, full-length membrane-spanning receptor proteins. Thus, competition between or interference by the products of the two alleles, rather than gene dosage of the resistant Col-0 allele, might explain the incomplete dominance of natural *RFO* alleles. Because physical interactions between RLKs and a RLP are critical for signaling in plants [28], genetic interaction between *RFO1* and *RFO2* might be evidence for the direct interaction of the corresponding RFO1 RLK and RFO2 RLP. However, because resistance is a complex phenotype, involving processes that occur at different sites in the host and at different times in the infection cycle, the observed genetic interaction might reveal the priority of *RFO1* before *RFO2* without direct interaction. In addition, when neither resistant nor susceptible allele is the null allele, interpretation of genetic interaction is ambiguous. For example, results with *rfo1* clearly implicate *RFO1-C* in the *RFO1*-*RFO2* interaction, however, it remains unclear whether *RFO1-C* suppresses resistance of *RFO2-T* or enhances resistance of *RFO2-C.*

Routine QTL analysis in *A. thaliana* is limited to natural traits that distinguish parents of existing RI populations; otherwise, the generation of new RI lines represents a substantial investment of time and effort [4,5]. In the meantime, there is an increasing availability of whole genome sequence, from the 1001 Genomes Project for example, that makes the sequence diversity in hundreds of *Arabidopsis* accessions accessible. As shown here, the mapping of traits that distinguish any two sequenced accessions, including mutant genotypes, can be conceive and complete in six months using BC_1_ populations.

Mapping in BC_1_ populations can be a routine procedure when genotyping is efficient, accessible and economical. In this regard, available whole genome sequence from the 1001 Genomes Project was an invaluable resource for identifying accession-specific polymorphisms [29]. Primer sequences for Col-0-specific dominant markers were readily selected from genome sequence reported to be polymorphic in Col-0 and Ty-0. In the same way, dominant markers could be designed to distinguish any two sequenced accessions. In fact, we have reused most of the Col-0-specific markers for genotyping BC_1_ populations from crosses between Col-0 and accessions Zdr-1 or Kondara (unpublished observation).

The methodology for genotyping was designed with efficiency and economy in mind. Starting from crude leaf preparations, multiplex PCR DNA of 40 dominant markers was amplified by just three sets of multiplex PCR primers and visualized using standard agarose-gel electrophoresis. The 200 FOC1-infected BC_1_ hybrids were genotyped genome-wide with little more than six 96-well plates of PCR samples. Markers in a multiplex PCR sample appeared as a ladder of bands in agarose gels when all markers were present. Because annealing of marker primers distinguished the Col-0 and Ty-0 genotypes, markers could be arbitrarily assigned sequence lengths that appeared as regularly spaced bands in agarose gels.

Results obtained with dominant markers were as reliable as results from codominant SSLP markers [1]. No unforeseen PCR products were amplified when as many as 14 primer pairs were combined in multiplex PCR, and no primer pairs that were confirmed singly subsequently failed when combined with other primer pairs.

In theory, an RI population has roughly twice as many crossovers as a BC_1_ population [30]. However, the additional recombination in RI lines remains largely unappreciated unless a high density of DNA markers are used to genotype RI lines [31]. During the inbreeding cycles that generate RI lines, crossovers tend to accumulate at linked sites, and thus recombination in RI lines has the appearance of negative interference. High-resolution analysis of breakpoints in 98 Col-0/L*er* RI lines found, for example, that 17 percent of intervals between crossovers contained just one gene [32].

For genome-wide linkage analysis in BC_1_ populations, 40 evenly spaced markers should be sufficient to capture most recombination. As already mentioned, just one set of homologous chromosomes in BC_1_ hybrids is recombinant. With an average marker separation of 15 cM, I estimate that just seven percent of crossovers went undetected in the BC_1_ populations because approximately three quarters of expected double crossovers in marker intervals would be suppressed by positive interference. In addition, I took advantage of significantly higher recombination in male meiosis (as compared to female meiosis) in *A. thaliana* when generating the BC_1_ hybrids [33]: Ty-0 was the female parent in the backcross while the F_1_ hybrid, which was the source of recombinant chromosomes, was the male parent.

Number of crossovers, or amount of recombination, has little bearing on whether a lone QTL is detected [4,13]. Rather, recombination frequency affects the resolution of map position of a QTL, and less recombination would more poorly resolve multiple QTLs in proximity on a chromosome. The detection of two or more linked loci could be suppressed if the loci that remain unresolved express opposing effects on a trait. Indeed, an example of two opposing QTLs for growth rate within an interval of 210 kbp has been reported in Col-0/L*er* recombinants [34].

QTL mapping is just the first step in the identification and characterization of the genes underlying traits. In this regard, mapping in BC_1_ populations is also advantageous because individual (or specific combinations of) QTLs can immediately be reevaluated and fine-mapped in progeny of selfed BC_1_ hybrids. Even after a potentially lethal test, such as resistance to FOM, I was able to collect seeds from 144 of 236 tested C-T BC_1_ hybrids. Although half of the genome in BC_1_ hybrids was heterozygous, on average, seeds were collected from 16 BC_1_ hybrids that were largely homozygous Ty-0 and heterozygous in just four or fewer chromosomal intervals representing 30 percent or less of the genome. *RFO* QTLs in these heterozygous intervals would again segregate in progeny.

## Conclusions

Genome-wide mapping of quantitative Fusarium wilt resistance was expeditious and reproducible in BC_1_ recombinant populations of *A. thaliana*. In two independent BC_1_ populations, resistance to FOM was associated with QTLs *RFO3*, *RFO4* and *RFO5*. Because the resistance of *RFO1*, *RFO2* and *RFO6* was absent in the BC_1_ population that included *rfo1*, the major effect and epistatic interactions of *RFO1* were solely attributed to At1g79670, the gene disrupted in *rfo1*. In a third BC_1_ population, resistance to a second pathogen FOC1 was instead associated with *RFO7*, a new major effect QTL. Pathogen-specific *RFO* QTLs were largely responsible for resistance to the two pathogens, FOM and FOC1.

## Methods

### Growing *A. thaliana*

Seeds of Ty-0 (CS6768) and *rfo1* (Salk_077975) were obtained from the Arabidopsis Biological Resource Center. Seeds were surface-sterilized in 10% household bleach and 0.1% Triton X-100 for 15 min, rinsed 3 times in sterile water. Seeds were sown on peat pellets (Jiffy-730, Grower’s Solution Inc., Cookeville, TN) or first germinated on plant nutrient agar (PNA) before transplanting [1]. Plants were arrayed in flats (1′ × 2′) 5 rows by 10 columns and designated: first by flat (1 through 6), second by row (A to E) and third by column (1 through 10). Plants were grown under medium intensity cool white fluorescent lighting (100 to 140 μmoles m^-2^ sec^-1^) for a 12-hr daylength at 25 to 28°C and irrigated with water or fertilizer (PlugCarePlus, Greencare Fertilizers, Inc., Kankakee, IL).

### Infection with *F. oxysporum*

*Fusarium oxysporum* forma specialis *conglutinans* race 1 (FOC1, isolate 777) and *Fusarium oxysporum* forma specialis *matthioli* (FOM, isolate 726) are from P.H. Williams by way of H.C. Kistler [3,35]. *F. oxysporum* cultures were stored at -80°C in 50% glycerol, grown on Czapek Dox medium (Oxoid Ltd., Hampshire, England) and harvested as described in [1]. Starting with an excess of 3-week old plants, 200 C-T BC_1_, 25 Col-0 and 25 Ty-0 plants with comparable sizes were infected with FOC1; and, 190 *r*-T BC_1_, 25 *rfo1*, and 15 Ty-0 plants were infected with FOM. Plants were irrigated with an excess of washed conidia (2 × 10^6^ conidia mL^-1^). The FOM-infected *r*-T population was scored 11 days post infection (dpi) for three early symptoms: (i) stunting of leaves, (ii) leaf epinasty and (iii) anthocyanin accumulation, using a graduated scale of 1 (severe) to 4 (unaffected). At 18 and 23 dpi, infected plants were scored using a health index (HI), which is the same as the disease index (DI) in [1], ranging from 0 (dead plants) to 5 (unaffected plants) in intervals of 0.5. The FOC1-infected C-T population was similarly scored on 10, 13 and 16 dpi. At the final time point, plants were rank ordered: For the FOC1-infected C-T population, each flat of 40 plants was ranked separately, from 1 (most susceptible) to 40 (most resistant); and, for the FOM-infected *r*-T BC population, all 190 plants were ranked together, from 1 (most susceptible) to 190 (most resistant). Infection and scoring of the FOM-infected C-T BC population is described in [1]. (See Additional file 8: for spreadsheets with phenotypic data of all three BC_1_ populations).

### Genotyping with CHR markers

In proportion to physical and genetic lengths of chromosomes in The Arabidopsis Information Resource (TAIR, http://www.arabidopsis.org), 10, 6, 8, 7 and 9 CHR markers were distributed on chromosomes 1, 2, 3, 4 and 5, respectively. On each chromosome, two markers were placed close to the telomeres, and nucleotide positions for remaining markers were spaced at regular intervals in the reference Col-0 sequence of TAIR10.

At the approximate nucleotide positions of markers, marker sequences were reference sequences that were classified as highly diverged, or "unsequenced", in whole genome sequencing of Ty-0 ( http://signal.salk.edu/atg1001). Appropriate pairs of primer sequences were selected in the highly diverged reference sequence using Primer3Plus software, according to recommendations of the QIAGEN Multiplex PCR Handbook (Qiagen Inc., Valencia, CA) [36]. DNA products of 13, 13 or 14 markers were simultaneously amplified by multiplex PCR using three sets of PCR primers. Each set of multiplex PCR primers were designed to give a logarithmic progression of DNA product sizes, ranging from 200 bp to 650 bp in length, which gave regular spacing of marker bands when products were size-separated by agarose gel electrophoresis. Primer sequences and genomic locations of PCR primers are in Additional file 9: Table S5. Sizes and order of DNA products for each set of primers are in Additional file 10: Table S6. PCR amplification was performed using the QIAGEN Multiplex PCR kit according to the protocol for microsatellite loci. A reaction volume of 5 μL included 1 μL crude leaf DNA preparation, 2.5 μL 2× QIAGEN Master Mix, 1 μL 10× primer mix (containing 2 μM of each oligonucleotide primer), and 1.5 μL water. Amplified PCR products were separated by gel electrophoresis in 2% agarose. Crude leaf DNA preparations were prepared according to [37]. See Additional file 8: for spreadsheets with genotypic data for all markers in all BC_1_ populations. Genotypic data for simple sequence length polymorphism (SSLP) C4H, from a prior study [1], replaced CHR2.4s in the analysis of the FOM-infected C-T population.

Genetic distances between markers were calculated using the Kosambi mapping function [13]. Genetic linkage supported the presumed physical linkage of markers in the three BC_1_ populations. Linkage data for markers in the three mapping populations are provided in Additional file 3: Table S1, Additional file 4: Table S2 and Additional file 7: Table S4.

### Testing association of wilt resistance

A BC_1_ population of *n* plants was rank ordered according to HI scores, from 1 (most susceptible) to *n* (most resistant). Ranking gave priority to later HI scores over earlier HI scores. Rank distributions of the two possible genotypes C/T and T/T were compared using the Mann-Whitney (M-W) test, the results of which were expressed as a standardized statistic (*Z*), the standard deviation units separating the mean ranks of the two genotypes. (See Additional file 11: Table S7 with values of *Z* at CHR markers in the three BC_1_ populations.) For a major effect QTL, threshold values of *Z* for the three BC_1_ populations were determined by permutation tests with 10,000 trials [18]. From the distribution of highest *Z* values in trials, the threshold value of *Z* at *p* = 0.01 (*Z*_0.01_) was 3.86; and, *Z*_0.05_ was 3.36; and, *Z*_0.20_ was 2.80. In *r-*T population, *Z*_0.01_ = 3.73; and, *Z*_0.05_ = 3.27; and, *Z*_0.20_ = 2.77. In FOC1-infected C-T population, *Z*_0.01_ = 3.63; and, *Z*_0.05_ = 3.16; and, *Z*_0.20_ = 2.68. Probability threshold values of *Z* were also determined for QTLs conditioned by genotype at a major QTL [20]. For FOC1-infected C-T population, the M-W test was performed on subpopulations that were either genotype T/T (*Z*_0.01_ = 3.54; and, *Z*_0.05_ = 3.10; and, *Z*_0.20_ = 2.67) or C/T (*Z*_0.01_ = 3.51; and, *Z*_0.05_ = 3.08; and, *Z*_0.20_ = 2.63) at *RFO7*-linked CHR5.7. For FOM-infected plants, subpopulations were tested that were either T/T (for *r*-T population, *Z*_0.01_ = 3.56; and, *Z*_0.05_ = 3.13; and, *Z*_0.20_ = 2.67; and, for C-T population, *Z*_0.01_ = 3.55; and, *Z*_0.05_ = 3.12; and, *Z*_0.20_ = 2.66) or C/T (for *r*-T population, *Z*_0.01_ = 3.52; and, *Z*_0.05_ = 3.12; and, *Z*_0.20_ = 2.64; and, for C-T population, *Z*_0.01_ = 3.88; and, *Z*_0.05_ = 3.34; and, *Z*_0.20_ = 2.81) at *RFO1*-linked CHR1.9.

## Competing interests

The author declares that he has no competing interests.

## Supplementary Material

Additional file 1: Figure S1 Genetic map of CHR markers in FOM-infected *r*-T population. Click here for file

Additional file 2: Figure S2 Genetic map of SSLP and CHR markers in FOM-infected C-T population. Click here for file

Additional file 3: Table S1 Linkage of CHR markers in the FOM-infected *r-*T BC_1_ population. Click here for file

Additional file 4: Table S2 Linkage of SSLP and CHR markers in FOM-infected C-T BC_1_ population/. Click here for file

Additional file 5: Table S3 Observed and expected double crossovers in BC_1_ populations. Click here for file

Additional file 6: Figure S3 Genetic map of CHR markers in FOC1-infected C-T population. Click here for file

Additional file 7: Table S4 Linkage of CHR markers in FOC-infected C-T BC_1_ population. Click here for file

Additional file 8 **Genotypes and phenotypes of BC**_**1 **_**hybrids in three *F. oxysporum*-infected populations.** Click here for file

Additional file 9: Table S5 Sequence and location of PCR primers. Click here for file

Additional file 10: Table S6 Expected PCR products of three sets of multiplex markers. Click here for file

Additional file 11: Table S7 *Z* of CHR markers in the three BC_1_ populations. Click here for file
