# Supplementary material for: Routine mapping of Fusarium wilt resistance in BC1 populations of Arabidopsis thaliana
Source: BMC Plant Biol. 2013 Oct 30;13:171. doi: 10.1186/1471-2229-13-171 (PMC3819736; doi:10.1186/1471-2229-13-171)
Supplement: Additional file 2: Figure S2 — Genetic map of SSLP and CHR markers in FOM-infected C-T population. [file 1471-2229-13-171-S2.pdf]

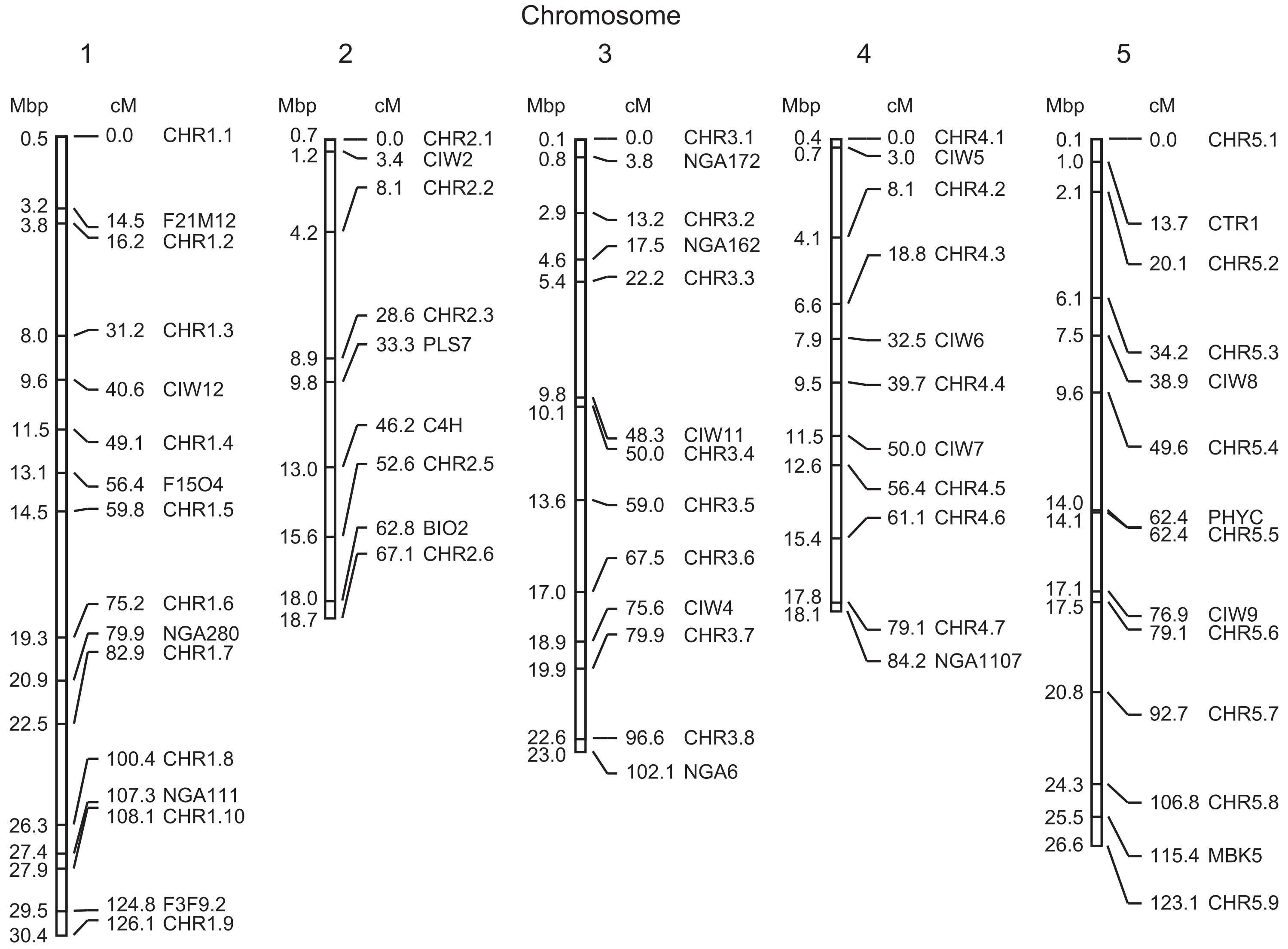

Figure S2. Genetic map of SSLP and CHR markers in FOM-infected C–T population

Corresponding nucleotide positions in megabasepairs (Mbp, to the left) and genetic positions in centiMorgans (cM, to the right) of 24 SSLP and 39 CHR markers on the five *Arabidopsis* chromosomes (vertical bars) are shown. Nucleotide positions are from the TAIR10 reference sequence. Genetic distances between markers were calculated using the Kosambi mapping function and recombination frequencies in the FOM-infected C-T BC_1 population.
