# Supplementary material for: Routine mapping of Fusarium wilt resistance in BC1 populations of Arabidopsis thaliana
Source: BMC Plant Biol. 2013 Oct 30;13:171. doi: 10.1186/1471-2229-13-171 (PMC3819736; doi:10.1186/1471-2229-13-171)
Supplement: Additional file 3: Table S1 — Linkage of CHR markers in the FOM-infected r-T BC1 population. [file 1471-2229-13-171-S3.pdf]

Table S1. Linkage of CHR markers in the FOM-infected *r*-T BC_1 population

Chromosome 1			Chromosome 2			Chromosome 3			Chromosome 4			Chromosome 5		
Marker[a]	Rf[b]	cM[c]	Marker	Rf	cM	Marker[c]	Rf	cM	Marker	Rf	cM	Marker	Rf	cM
CHR1.1	—	0.0	CHR2.1	—	0.0	CHR3.1	—	0.0	CHR4.1	—	0.0	CHR5.1	—	0.0
CHR1.2	0.193	19.3	CHR2.2r	0.107	10.7	CHR3.2	0.206	20.6	CHR4.2	0.107	10.7	CHR5.2m	0.264	26.4
CHR1.3	0.237	43.1	CHR2.3o	0.271	37.8	CHR3.3	0.118	32.4	CHR4.3	0.124	23.0	CHR5.3	0.074	33.8
CHR1.4	0.169	60.0	CHR2.4s	0.212	59.0	CHR3.4	0.257	58.1	CHR4.4	0.124	35.4	CHR5.4	0.199	53.8
CHR1.5	0.112	71.2	CHR2.5	0.096	68.6	CHR3.5	0.107	68.8	CHR4.5	0.140	49.4	CHR5.5	0.101	63.9
CHR1.6	0.146	85.9	CHR2.6	0.251	93.6	CHR3.6	0.058	74.6	CHR4.6	0.048	54.2	CHR5.6	0.096	73.5
CHR1.7n	0.112	97.1				CHR3.7	0.129	87.5	CHR4.7	0.278	82.0	CHR5.7	0.129	86.4
CHR1.8	0.224	119.5				CHR3.8	0.112	98.7				CHR5.8	0.129	99.3
CHR1.10	0.112	130.8										CHR5.9m	0.237	123.1
CHR1.9	0.231	153.9												

[a] DNA markers are described in Methods.
[b] Recombination frequency is between marker and above marker.
[c] Map position is in centiMorgans.
