# Supplementary material for: Routine mapping of Fusarium wilt resistance in BC1 populations of Arabidopsis thaliana
Source: BMC Plant Biol. 2013 Oct 30;13:171. doi: 10.1186/1471-2229-13-171 (PMC3819736; doi:10.1186/1471-2229-13-171)
Supplement: Additional file 4: Table S2 — Linkage of SSLP and CHR markers in FOM-infected C-T BC1 population/. [file 1471-2229-13-171-S4.pdf]

Table S2. Linkage of SSLP and CHR markers in FOM-infected C-T BC_1 population

Chromosome 1			Chromosome 2			Chromosome 3			Chromosome 4			Chromosome 5		
Marker[a]	Rf[b]	cM[c]	Marker	Rf	cM	Marker	Rf	cM	Marker	Rf	cM	Marker	Rf	cM
CHR1.1	—	0.0	CHR2.1	—	0.0	CHR3.1	—	0.0	CHR4.1	—	0.0	CHR5.1	—	0.0
F21M12	0.150	15.0	CIW2	0.034	3.4	NGA172	0.039	3.9	CIW5	0.030	3.0	CTR1	0.140	14.0
CHR1.2	0.017	16.7	CHR2.2	0.047	8.1	CHR3.2	0.095	13.4	CHR4.2	0.051	8.1	CHR5.2	0.064	20.5
CHR1.3	0.154	32.1	CHR2.3	0.218	29.9	NGA162	0.043	17.7	CHR4.3	0.109	19.0	CHR5.3	0.145	35.0
CIW12	0.095	41.6	PLS7	0.047	34.6	CHR3.3	0.047	22.4	CIW6	0.140	33.0	CIW8	0.047	39.7
CHR1.4	0.086	50.2	C4H	0.131	47.8	CIW11	0.289	51.3	CHR4.4	0.073	40.3	CHR5.4	0.109	50.5
F15O4	0.073	57.6	CHR2.5	0.064	54.2	CHR3.4	0.017	53.0	CIW7	0.104	50.7	PHYC	0.131	63.7
CHR1.5	0.034	61.0	BIO2	0.104	64.6	CHR3.5	0.091	62.1	CHR4.5	0.064	57.2	CHR5.5	0.000	63.7
CHR1.6	0.159	76.9	CHR2.6	0.043	68.9	CHR3.6	0.086	70.7	CHR4.6	0.047	61.9	CIW9	0.150	78.6
NGA280	0.047	81.6				CIW4	0.082	78.9	CHR4.7	0.188	80.7	CHR5.6	0.021	80.8
CHR1.7	0.030	84.6				CHR3.7	0.043	83.2	NGA1107	0.051	85.8	CHR5.7	0.140	94.8
CHR1.8	0.183	102.9				CHR3.8	0.173	100.5				CHR5.8	0.145	109.3
NGA111	0.069	109.8				NGA6	0.056	106.1				MBK5	0.086	117.9
CHR1.10	0.009	110.6										CHR5.9	0.078	125.7
F3F9.2	0.173	128.0												
CHR1.9	0.013	129.2												

[a] DNA markers are described in Methods.
[b] Recombination frequency is between marker and above marker.
[c] Map position is in centiMorgans.
