# Supplementary material for: Routine mapping of Fusarium wilt resistance in BC1 populations of Arabidopsis thaliana
Source: BMC Plant Biol. 2013 Oct 30;13:171. doi: 10.1186/1471-2229-13-171 (PMC3819736; doi:10.1186/1471-2229-13-171)
Supplement: Additional file 5: Table S3 — Observed and expected double crossovers in BC1 populations. [file 1471-2229-13-171-S5.pdf]

Table S3. Observed and expected double crossovers in BC_1 populations

	Observed (expected) double crossovers[a] Chromosome[b]					
BC population	1	2	3	4	5	All
C-T (FOC)[c]	5 *(25)*	4 *(14)*	3 *(18)*	1 *(14)*	10 *(25)*	23 (*96*)
C-T (FOM)[d]	6 *(30)*	4 *(16)*	3 *(24)*	2 *(19)*	9 *(36)*	24 (*125*)
r-T (FOM)[e]	13 *(38)*	8 *(22)*	4 *(19)*	7 *(12)*	5 *(22)*	37 (*113*)

[a] Crossovers in adjacent marker intervals were counted as double crossovers. The expected frequency of double crossovers is the product of recombination frequencies of adjacent interval, and the number of expected double crossovers is the product of double crossover frequency and the number of plants in the population.
[b] There are 35 marker intervals between 40 markers: 9, 5, 7, 6 and 8 on chromosomes 1, 2, 3, 4 and 5.
[c] Among 200 FOC-infected Col-0 x Ty-0 BC plants
[d] Among 234 FOM-infected Col-0 x Ty-0 BC plants
[e] Crossovers among 190 FOM-infected *rfo1* (Col-0) x Ty-0 BC plants
