# Supplementary material for: Routine mapping of Fusarium wilt resistance in BC1 populations of Arabidopsis thaliana
Source: BMC Plant Biol. 2013 Oct 30;13:171. doi: 10.1186/1471-2229-13-171 (PMC3819736; doi:10.1186/1471-2229-13-171)
Supplement: Additional file 6: Figure S3 — Genetic map of CHR markers in FOC1-infected C-T population. [file 1471-2229-13-171-S6.pdf]

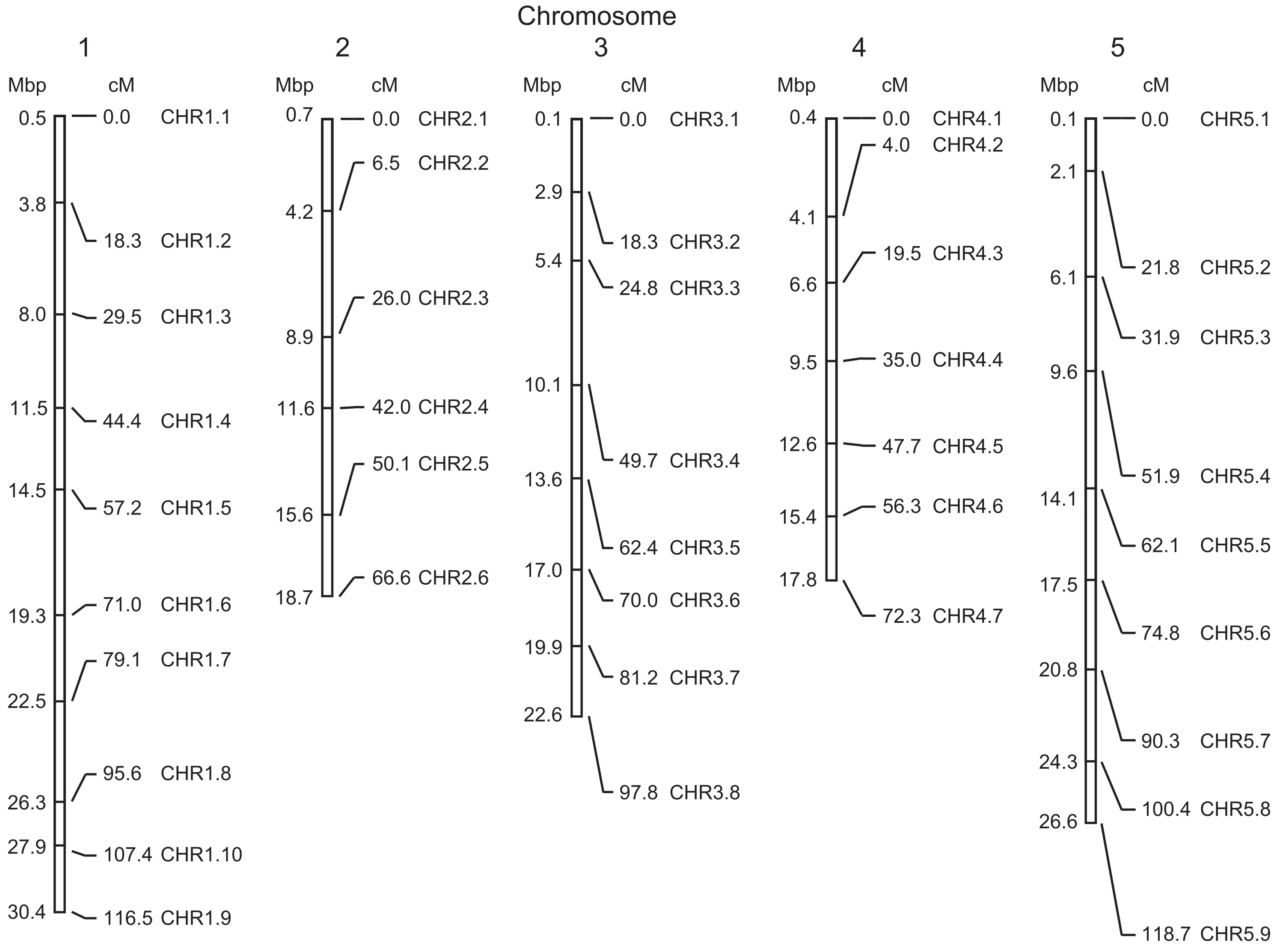

Figure S3. Genetic map of CHR markers in FOC1-infected C–T population

Corresponding nucleotide positions in megabasepairs (Mbp, to the left) and genetic positions in centiMorgans (cM, to the right) of 40 CHR markers on the five *Arabidopsis* chromosomes (vertical bars) are shown. Nucleotide positions are from the TAIR10 reference sequence. Genetic distances between markers were calculated using the Kosambi mapping function and recombination frequencies in the FOC1-infected C-T BC_1 population.
