# Supplementary material for: Routine mapping of Fusarium wilt resistance in BC1 populations of Arabidopsis thaliana
Source: BMC Plant Biol. 2013 Oct 30;13:171. doi: 10.1186/1471-2229-13-171 (PMC3819736; doi:10.1186/1471-2229-13-171)
Supplement: Additional file 7: Table S4 — Linkage of CHR markers in FOC-infected C-T BC1 population. [file 1471-2229-13-171-S7.pdf]

Table S4. Linkage of CHR markers in FOC-infected C-T BC_1 population

Chromosome 1			Chromosome 2			Chromosome 3			Chromosome 4			Chromosome 5		
Marker[a]	Rf[b]	cM[c]	Marker	Rf	cM	Marker	Rf	cM	Marker	Rf	cM	Marker	Rf	cM
CHR1.1	—	0.0	CHR2.1	—	0.0	CHR3.1	—	0.0	CHR4.1	—	0.0	CHR5.1	—	0.0
CHR1.2	0.183	18.3	CHR2.2r	0.065	6.5	CHR3.2	0.183	18.3	CHR4.2	0.040	4.0	CHR5.2m	0.218	21.8
CHR1.3	0.112	29.5	CHR2.3o	0.194	26.0	CHR3.3	0.065	24.8	CHR4.3	0.155	19.5	CHR5.3	0.101	31.9
CHR1.4	0.149	44.4	CHR2.4s	0.160	42.0	CHR3.4	0.249	49.7	CHR4.4	0.155	35.0	CHR5.4	0.200	51.9
CHR1.5	0.128	57.2	CHR2.5	0.081	50.1	CHR3.5	0.128	62.4	CHR4.5	0.128	47.7	CHR5.5	0.101	62.1
CHR1.6	0.138	71.0	CHR2.6	0.166	66.6	CHR3.6	0.076	70.0	CHR4.6	0.086	56.3	CHR5.6	0.128	74.8
CHR1.7n	0.081	79.1				CHR3.7	0.112	81.2	CHR4.7	0.160	72.3	CHR5.7	0.155	90.3
CHR1.8	0.166	95.6				CHR3.8	0.166	97.8				CHR5.8	0.101	100.4
CHR1.10	0.117	107.4										CHR5.9m	0.183	118.7
CHR1.9	0.091	116.5												

[a] DNA markers are described in Methods.
[b] Recombination frequency is between marker and above marker.
[c] Map position is in centiMorgans.
