# Supplementary material for: Routine mapping of Fusarium wilt resistance in BC1 populations of Arabidopsis thaliana
Source: BMC Plant Biol. 2013 Oct 30;13:171. doi: 10.1186/1471-2229-13-171 (PMC3819736; doi:10.1186/1471-2229-13-171)
Supplement: Additional file 9: Table S5 — Sequence and location of PCR primers. [file 1471-2229-13-171-S9.pdf]

Table S5. Sequence and location of PCR primers

Primer name	Nucleotide sequence	Chromosome	Nucleotide[a]
CHR1.1-F	tttgttaaccaacatgcaattgcgaatc	1	522998
CHR1.1-R	tctcgtgagttaatatgaaaacttgtttattcc	1	523187
CHR1.2-F	gctttagcttggtggtaacggatgagg	1	3774539
CHR1.2-R	tgtgttgtagtctggtggtgaaagctg	1	3775015
CHR1.3-F	ttaaatcaggctggccctaatgccttt	1	7969890
CHR1.3-R	gtggattttgcagtgctagttccctca	1	7970182
CHR1.4-F	gatggccactcttcgggacggaaagat	1	11486951
CHR1.4-R	ttttgcttgtcaatgataccgctaggc	1	11487548
CHR1.5-F	gtgtctagccggcagaaaatcgtgttg	1	14544932
CHR1.5-R	tgcaggcttcaattcgtgagcttagtc	1	14545134
CHR1.6-F	tgccaatactacctccaactccaatga	1	19265859
CHR1.6-R	tgcgatgactggtaaccgaactcttca	1	19266379
CHR1.7n-F	aaatccgaagagaaaaccggttgaaca	1	22502498
CHR1.7n-R	cctaatctcagcgaataacacaaaagaccaag	1	22502752
CHR1.8-F	aggtggctcaaacaattcagacatcttca	1	26280217
CHR1.8-R	tcatgcagattgacaaaatctccctctttc	1	26280547
CHR1.9-F	gaaaaggagatgccgtttcgtccctac	1	30425560
CHR1.9-R	caaagtcggtttcgatttgggttttgt	1	30426060
CHR1.10-F	aaacgctggaaatgaacccactttgct	1	27919865
CHR1.10-R	catctgaagcaccataaccaccaccag	1	27920045
CHR2.1-F	ttcagtagatcgagcttgcaaatggaga	2	748923
CHR2.1-R	cggacatgagtggatgattaagttgacc	2	749118
CHR2.2r-F	agaagaggagaagacgaaatgtttggaag	2	4191023
CHR2.2r-R	cagaagcaaaagcagcgcagccagagt	2	4191264
CHR2.3o-F	cgagttttcaaggtatctttttgccgacat	2	8928456
CHR2.3o-R	gtggctaacgatgaacattgtccaagc	2	8928721
CHR2.4r-F	aggtgaatgtcgagctaagtttgtgtgc	2	11590986
CHR2.4r-R	catcttgcacaactacagcatccgttg	2	11591416
CHR2.5-F	gctcctgctgttcacgagccttctact	2	15602732
CHR2.5-R	taaccaacatgtcttaaaacttgccgaacc	2	15603022
CHR2.6-F	caaacgatggtcgagaccctaatggag	2	18659800
CHR2.6-R	gggtggcaatcaaacttcctctctcct	2	18660187
CHR3.1-F	tgtcgctctcttctcaatggctatgtca	3	129269
CHR3.1-R	aagtttgtttgcgatgaggctcggtct	3	129591
CHR3.2-F	tggaagtattagtggcggatctacattgc	3	2867425
CHR3.2-R	aacccaaaacttgaactgaaaggtggcta	3	2867667
CHR3.3-F	tgatggagtgaaaaagcatgtccattg	3	5440460
CHR3.3-R	ctgaaagtataaccaaagcgcgagcaa	3	5440672
CHR3.4-F	ccccaccaattgaagacaaccatacaa	3	10116094
CHR3.4-R	tgccttatagacaggttggtactgaga	3	10116531
CHR3.5-F	ctcttgcaaccattacccattccgact	3	13614386
CHR3.5-R	ctaccaccgcgttagcttttgggatgt	3	13614935
CHR3.6-F	ccctgtctcaattgtttgctgagactg	3	17046915

CHR3.6-R	tgtcaatcctctgaaatcgtgaggaga	3	17047294
CHR3.7-F	ttctccggttaacttggcagatttcaga	3	19913874
CHR3.7-R	acgagtgacgaaaccgtcatctactga	3	19914149
CHR3.8-F	gtattggcgactccaaatcccattcac	3	22558313
CHR3.8-R	ctcctgtgatagcctcacccgcttct	3	22558677
CHR4.1-F	gcagcgtagatcaacgtttcagcacat	4	426075
CHR4.1-R	aaaagcgtaagggcatcggttcttcat	4	426396
CHR4.2-F	cctcttagtgtattactttcatgattcgtcagca	4	4104808
CHR4.2-R	tgcactttcaagtttagaataccatcgtgaca	4	4105277
CHR4.3-F	ctccatccacctacgggcaaagtctac	4	6591300
CHR4.3-R	atcataggatgggaagtttcgggagca	4	6591817
CHR4.4-F	tcggagtcaccatttgatccaagtttt	4	9530122
CHR4.4-R	tttgagcaaactctcatgtgacgcaac	4	9530746
CHR4.5-F	ttggttttgcatgtccagatttgtgag	4	12623968
CHR4.5-R	agtttgccgaagaagaagaacgaacaag	4	12624534
CHR4.6-F	tgatgtgaatcctgcaatctccttttcg	4	15363337
CHR4.6-R	aaaagtttgggccaacataacgatgctc	4	15363688
CHR4.7-F	cacatatgtgctcgtctacaattacatg	4	17772273
CHR4.7-R	tgacgtttcatcataatctttcccgcc	4	17772492
CHR5.1-F	gactgtttctctcatcccgaagtcgtc	5	136060
CHR5.1-R	ctcggagacgatgcgaacggtaatatc	5	136630
CHR5.2m-F	ctcccaagccacgacaagaccgtagta	5	2106256
CHR5.2m-R	ggtggagagaatgatagcgtggaggaa	5	2106475
CHR5.3-F	gagataaaagggaacccggaagctgtg	5	6075313
CHR5.3-R	tcaaggagttcccaaatgttcctgtca	5	6075771
CHR5.4-F	aaagcttgatgggaagttcgtgctttg	5	9615178
CHR5.4-R	tgaggctgggaactattgcctcttacg	5	9615409
CHR5.5-F	tgcatccggtgaaaaacaatcaacact	5	14106388
CHR5.5-R	aaaagctggctggtggtctccctctac	5	14106691
CHR5.6-F	ggcaagggaggtttcttccccttaat	5	17462820
CHR5.6-R	gcagctttagcaactgcatctgccttac	5	17463086
CHR5.7-F	gagtcaccttcttcagttggcctggaa	5	20821073
CHR5.7-R	atgaggagctttgcaggtcggatatga	5	20821421
CHR5.8-F	atggcacaatcaaccaaggaaaaggtg	5	24272565
CHR5.8-R	gtccagttgttgcaaccggcttcagt	5	24272963
CHR5.9m-F	tattcagaagtatagaggggtaggggg	5	26589095
CHR5.9m-R	ggtcaggctggcatgtgtgattgggtc	5	26589512

[a] Position of most 5' nucleotide in the Col-0 reference genome sequence.
