# Supplementary material for: Routine mapping of Fusarium wilt resistance in BC1 populations of Arabidopsis thaliana
Source: BMC Plant Biol. 2013 Oct 30;13:171. doi: 10.1186/1471-2229-13-171 (PMC3819736; doi:10.1186/1471-2229-13-171)
Supplement: Additional file 10: Table S6 — Expected PCR products of three sets of multiplex markers. [file 1471-2229-13-171-S10.pdf]

Table S6. Expected PCR products of three sets of multiplex markers

Set 1[a]		Set 2		Set 3	
Marker	Basepairs	Marker	Basepairs	Marker	Basepairs
CHR1.4	598	CHR5.1	571	CHR4.4	625
CHR3.5	550	CHR1.6	521	CHR4.5	567
CHR1.9	501	CHR1.2	477	CHR4.3	518
CHR5.3	459	CHR3.4	438	CHR4.2	470
CHR5.9m	418	CHR5.8	399	CHR2.4s	429
CHR3.6	380	CHR3.8	365	CHR2.6	388
CHR5.7	349	CHR1.8	331	CHR4.6	352
CHR3.1	323	CHR5.5	304	CHR4.1	322
CHR1.3	293	CHR3.7	276	CHR2.5	291
CHR5.6	267	CHR1.7n	255	CHR2.3o	266
CHR3.2	243	CHR5.4	232	CHR2.2r	240
CHR5.2m	220	CHR3.3	213	CHR4.7	220
CHR1.5	203	CHR1.1	190	CHR2.1	196
CHR1.10	181				

[a] Set of 13 or 14 DNA markers are simultaneously amplified by multiplex PCR.
