# Supplementary material for: Routine mapping of Fusarium wilt resistance in BC1 populations of Arabidopsis thaliana
Source: BMC Plant Biol. 2013 Oct 30;13:171. doi: 10.1186/1471-2229-13-171 (PMC3819736; doi:10.1186/1471-2229-13-171)
Supplement: Additional file 11: Table S7 — Z of CHR markers in the three BC1 populations. [file 1471-2229-13-171-S11.pdf]

Table S7. *Z* of CHR markers in the three BC_1 populations

Chromosome	Marker[a]	Z[b]			*RFO*[f]
		C/T-FOM[c]	*r*/T-FOM[d]	C/T-FOC[e]	
1	CHR1.1	2.25	2.75	1.03	
1	CHR1.2	***3.77***	2.44	0.34	
1	CHR1.3	***3.51***	1.87	-0.08	*RFO2*
1	CHR1.4	1.74	1.15	0.53	
1	CHR1.5	2.18	0.83	1.20	
1	CHR1.6	1.71	1.78	2.71	
1	CHR1.7n	***3.00***	2.22	2.79	
1	CHR1.8	***6.11***	1.86	2.73	
1	CHR1.10	***6.81***	1.20	2.56	
1	CHR1.9	***9.53***	1.84	2.54	*RFO1*
2	CHR2.1	0.34	-0.38	-0.62	
2	CHR2.2r	1.33	-0.03	-1.83	
2	CHR2.3o	1.52	1.13	-0.65	
2	CHR2.4s	2.54	1.40	-1.13	
2	CHR2.5	2.13	1.45	-0.49	
2	CHR2.6	1.00	2.08	-0.11	
3	CHR3.1	2.06	2.48	1.12	
3	CHR3.2	***4.38***	***4.87***	2.05	
3	CHR3.3	***4.25***	***6.91***	1.51	*RFO3*
3	CHR3.4	1.32	2.56	0.14	
3	CHR3.5	1.09	0.79	1.27	
3	CHR3.6	1.26	0.31	0.56	
3	CHR3.7	-0.68	0.77	-0.13	
3	CHR3.8	-1.02	0.55	0.32	
4	CHR4.1	0.49	***3.68***	-0.54	
4	CHR4.2	1.53	***5.63***	-0.15	*RFO4*
4	CHR4.3	1.01	***4.10***	1.18	
4	CHR4.4	0.64	***3.18***	1.59	
4	CHR4.5	0.38	2.29	1.76	
4	CHR4.6	0.92	1.65	1.37	
4	CHR4.7	1.31	0.95	1.07	
5	CHR5.1	0.08	1.43	1.10	
5	CHR5.2m	2.25	***4.49***	0.93	
5	CHR5.3	***2.83***	***4.83***	1.25	*RFO5*
5	CHR5.4	***3.53***	***3.89***	-1.31	
5	CHR5.5	***3.64***	***3.49***	-3.55	
5	CHR5.6	2.39	1.86	**-7.57**	*RFO6*
5	CHR5.7	0.29	-0.06	**-8.73**	*RFO7*
5	CHR5.8	0.00	-0.21	**-6.90**	
5	CHR5.9m	-1.02	-1.81	**-3.51**	

[a] Markers are described in Methods
[b] Standardized statistic *Z*: Significant values are in bold italics.
[c] From FOM-infected Col-0/Ty-0 BC population.
[d] From FOM-infected *rfo1*/Ty-0 BC population.
[e] From FOC-infected Col-0/Ty-0 BC population.
[f] Approximate position of *RFO* QTL
